# Combined dynamic nuclear polarization and electron paramagnetic resonance at 0.34 T to investigate electrochemical lithium deposition on copper

**DOI:** 10.1038/s41598-025-01107-x

**Published:** 2025-05-26

**Authors:** Vera Michaela Barysch, Beatrice Wolff, Matthias Streun, Peter Jakes, Peter Philipp Maria Schleker, Josef Granwehr

**Affiliations:** 1 Institute of Energy Technologies, Forschungszentrum Jülich GmbH, 52425 Jülich, Germany; 2 Institute of Technical and Macromolecular Chemistry, RWTH Aachen University, 52056 Aachen, Germany; 3 Institute of Technology and Engineering, Forschungszentrum Jülich GmbH, 52425 Jülich, Germany

**Keywords:** DNP, NMR, EPR, Lithium-ion batteries, Knight shift, Plating, Energy, Physical chemistry

## Abstract

Despite extensive research conducted on plating and dendrite formation in lithium batteries, the molecular formation processes are not yet fully understood. Electron paramagnetic resonance (EPR) sensitively detects metallic Li species but misses non-paramagnetic ones. Nuclear magnetic resonance (NMR) is chemically selective, yet exhibits low sensitivity under low-field conditions. Dynamic nuclear polarization (DNP) overcomes this by transferring electron spin polarization to nuclei. Here, correlative EPR and DNP-enhanced $^{7}\textrm{Li}$ NMR of lithium on copper is demonstrated using a custom setup operating at 0.34 T with a sweepable electromagnet. DNP experiments were conducted in pulsed mode to minimize sample heating. The resulting enhanced $^{7}\textrm{Li}$ NMR signal allows the observation of electrochemically deposited lithium on copper, harvested from a Cu vs. Li cell, with an enhancement $\epsilon >400$. By changing the magnetic field strength by a few Gauss, the saturation of the conduction EPR transition was varied, leading to an altered Knight shift of metallic $^{7}\textrm{Li}$. The corresponding change of the DNP-polarized $^{7}\textrm{Li}$ chemical shifts in the range from 240 ppm to 80 ppm allowed an indirect, saturation-based distinction of EPR species. Moreover, an enhancement $\epsilon$ by a factor of about 2 of the $^1$H signal from the surrounding electrolyte of electrochemically deposited lithium was observed, indicating the potential to investigate the solid–electrolyte interface (SEI). The setup employed a battery cell housing developed for EPR, demonstrating its suitability for *in operando* experiments in the future.

**Supplementary Information:**

The online version contains supplementary material available at 10.1038/s41598-025-01107-x.

## Introduction

Lithium-ion batteries (LIBs) play a crucial role in advancing sustainability, being integral to common devices such as portable electronic devices or electric vehicles. Furthermore, the shift towards sustainably produced energy increases the need for battery storage to compensate for the variable availability of electricity^1,2^. To further improve battery performance, using a lithium metal anode instead of graphite is a promising approach, as lithium exhibits the largest specific capacity and a low redox potential^3,4^. Additional energy density improvement is made possible by using zero-excess anodes, where Li is directly deposited on the current collector (‘anode free’). However, similar to LIBs, where plating can occur if lithium is deposited on the graphite surface instead of being intercalated^5,6^, lithium metal batteries (LMBs) also promote dendrite formation because Li does not deposit homogeneously on the surface. These can impose a severe safety risk by creating short-circuits in the battery. The formation, control, and avoidanceof these dendrites is still not fully understood, requiring various methods for investigation^7,8^. Moreover, the formation of electrochemically inactive, ‘dead’ lithium decreases the amount of available active lithium and is another contribution to capacity loss^9^.

One critical process regarding safety and lifetime of LIBs and LMBs is the formation of the solid–electrolyte interphase (SEI), which depends on many different parameters, for example on the electrolyte solvent or additives used^10^. The SEI is composed of a complex heterogeneous mixture of crystalline and soft constituents, including Li$_2$O, LiF, Li$_2$CO$_3$, polyolefins, and semicarbonates^9^. There is also a correlation between the thickness of the SEI and the lithium deposition/stripping process, but the precise determination of the layer thickness and composition remains a challenge^9^.

Nuclear magnetic resonance (NMR) and electron paramagnetic resonance (EPR) are two complementary magnetic resonance spectroscopy methods that allow monitoring chemical processes in batteries^11–18^. While NMR is highly selective, it is rather insensitive due to the low magnetic moment of nuclear spins. Dynamic nuclear polarization (DNP), where polarization from electron spins is transferred onto hyperfine-coupled nuclear spins, provides an approach to mitigate this limitation for nuclei in the vicinity of unpaired electrons. Overhauser first proposed the idea of saturating the EPR transitions of conduction electrons in metals to enhance the NMR signal by several orders of magnitude^19^. Experimental proof of Overhauser’s idea was achieved by Carver and Slichter by enhancing the $^{7}\textrm{Li}$ NMR signal of metallic lithium^20^. For optimal polarization of nuclei in metallic samples by DNP, the particle size should be smaller than the skin depth at the transition frequency of the electron spins^21^. For this reason, in their initial DNP experiment, Carver and Slichter dispersed lithium in mineral oil^20^. The relevant skin depths for this work were 1–2 ${\upmu \hbox {m}}$ for microwaves (mws) and several dozens of micrometers for radio frequency (RF) pulses^22,23^. The Overhauser DNP (ODNP) mechanism relies on electron-nuclear cross-relaxation. It requires a time-dependent hyperfine interaction (HFI) that changes stochastically. In liquids, such as nitroxide radical solutions, this occurs due to the stochastic motion of molecules. In contrast, in metals such as lithium, the HFI changes due to the movement of mobile electrons within the metal. The ODNP effect follows an incoherent pathway upon saturation of the allowed single-quantum (SQ) transition^24^. Afterwards, cross-relaxation occurs, which depends on the zero-quantum (ZQ) electron–nuclear transition probability $W_0$ and the double-quantum (DQ) electron–nuclear transition probability $W_2$. This process is particularly efficient for metals, where Fermi contact (FC) interactions dominate that only induce ZQ transitions.

For LIBs, DNP offers the opportunity to investigate Li metal and its environment by exploiting the intrinsic conduction electrons of the metal for polarization transfer towards the nuclei, without the need of additional polarizing agents^23^. Several studies have suggested a selective signal enhancement by DNP for the investigation of LIBs at high magnetic fields^25–27^. However, these investigations often involve cryogenic temperatures, additional polarizing agents, and/or magic angle spinning. These environments deviate from the actual conditions a battery experiences under realistic conditions during cycling. To study batteries, DNP-enhanced NMR measurements using an EPR spectrometer are a promising approach that has already been demonstrated in the literature in several variations^28–31^.

For LIBs, it has been shown that EPR can be used to investigate lithium intercalation and deposition processes, and that it can probe the onset of plating very sensitively^13,32,33^. To distinguish different morphologies, contrast was achieved from variations of the EPR linewidth and signal phase^34^. However, investigating the Li metal deposition on copper emerged to be more challenging due to the absence of narrow linewidths for small Li structures with good contact to the copper^35^. By correlation of EPR imaging with atomic force microscopy, regions with different degree of contact between Li and the underlying Cu foil could be identified^36^. It would be helpful if this information could be extracted directly from a magnetic resonance experiment.

Conduction EPR is monitoring Pauli paramagnetism^37^. It is, therefore, directly connected with the Knight shift that metallic Li nuclei experience, and the chemical shift can be altered as a function of the saturation of the conduction EPR transition^23^. For a homogeneous line, as it is commonly observed for conduction EPR, saturation can be expressed as the reduction of the longitudinal magnetization $M_z$ relative to its equilibrium value $M_0$,

1 $$\begin{aligned} \frac{M_z}{M_0} = \frac{1 + \Omega ^2 T_2^2}{1 + a + \Omega ^2 T_2^2} \, , \end{aligned}$$

where $\Omega$ is the frequency offset from exact resonance, $a = \omega _1^2 T_1 T_2$ is the continuous-wave (CW) saturation parameter, $T_1$ is the longitudinal and $T_2$ is the transverse relaxation time constant, and $\omega _1$ is the amplitude of the irradiated mw field. For metallic Li, it was reported that at least 75 % of the Knight shift is caused by Pauli paramagnetism and is removed upon saturation of the EPR transition^38^. Therefore, two Li species may become resolvable in the DNP-enhanced NMR spectrum if they experience significantly different relaxation times. In the case of strong saturation, $a \gg 1$, the Overhauser DNP enhancement $\epsilon$ for Li metal becomes proportional to $M_z/M_0$ to a good approximation, since it is generally found that $T_1 \approx T_2$. Then the $\Omega$ dependence of $\epsilon$ or, complementarily, the Knight shift can be used to estimate $\omega _1$.

In this work, a low-field DNP setup operating at X-band mw frequencies for battery applications is developed, adapted to accommodate an EPR *in operando* cell housing described by Niemöller et al.^11^ An RF coil is devised, which is tested for its EPR compatibility as well as its suitability for NMR experiments on $^{7}\textrm{Li}$ and $^{1}\textrm{H}$. The necessity for pulsing of the mw field is examined, in order to minimize sample heating and decomposition caused by the mw irradiation. With this setup, lithium deposition on a copper current collector is investigated *post mortem* via conduction DNP-enhanced $^{7}\textrm{Li}$ NMR measurements. Possibilities to employ the Knight shift as an additional parameter for NMR contrast are explored. Moreover, DNP enhancement of $^1$H NMR spectra of proton-containing species in the immediate vicinity of electrochemically deposited lithium is assessed.

## Results and discussion

### DNP setup

**Fig. 1:**
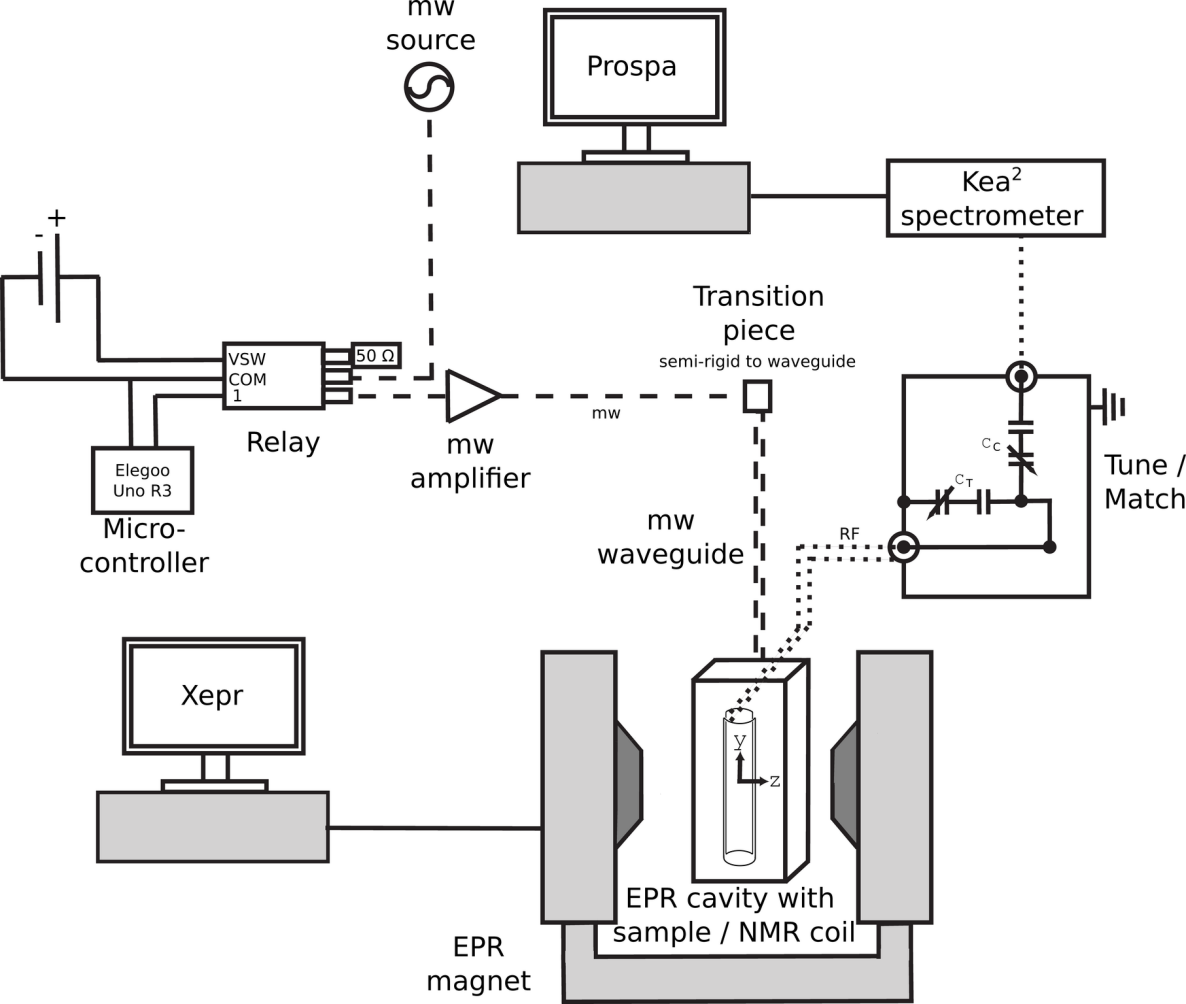
Schematic of the DNP setup. Full lines indicate electronic cables for powering, control and data transmission, dashed lines indicate the mw path, and dotted lines the RF path. The mw source was operated continuously. A relay was used to switch its output between a high-power amplifier and a 50 $\Omega$ load to facilitate pulsed sample irradiation. It was controlled by the Common (COM) and Voltage Switch (VSW) pins. The tune/match box on the right incorporated fixed and adjustable tuning and matching capacitors ($C_\textrm{T}$ and $C_\textrm{C}$, respectively), and a coil that was placed outside the box.

The setup consisted of three main parts: (i) an X-band EPR spectrometer (Bruker ElexSys E-540), which also provided the electromagnet used for all experiments, and an EPR cavity resonator (Bruker ER 4103TM), which was also used for DNP; (ii) a low-field NMR spectrometer console (Magritek Kea$^2$) connected to a custom resonant circuit; and (iii) a DNP channel with a mw source (Keysight Technologies N5173B, maximum power of 80 mW) whose output could be switched between a custom amplifier board^39^ with a maximum output power of 15.8 W followed by the mw resonator or a 50 $\Omega$ load for pulsed DNP operation (see Methods for more details). A schematic overview for DNP operation of the setup is provided in Fig. 1.

#### Coil geometry

To identify an NMR coil that was compatible with the EPR resonator modes, different coil geometries and copper wire alignments were tested with respect to their impact on the EPR resonance frequency and *Q*-factor. Horizontally aligned wire was found not to be compatible with the resonator mode, unless it was positioned at least 2 cm from the resonator center. Due to the horizontally aligned wire, regular solenoid or Helmholtz coils were ruled out. On the other hand, wires crossing the resonator vertically did not reduce its *Q*-factor as much. Therefore, a saddle coil geometry was selected to allow only vertically oriented wire in the resonator. A height of 4.5 cm was chosen to place the horizontal wire loops outside the cavity.

**Fig. 2:**
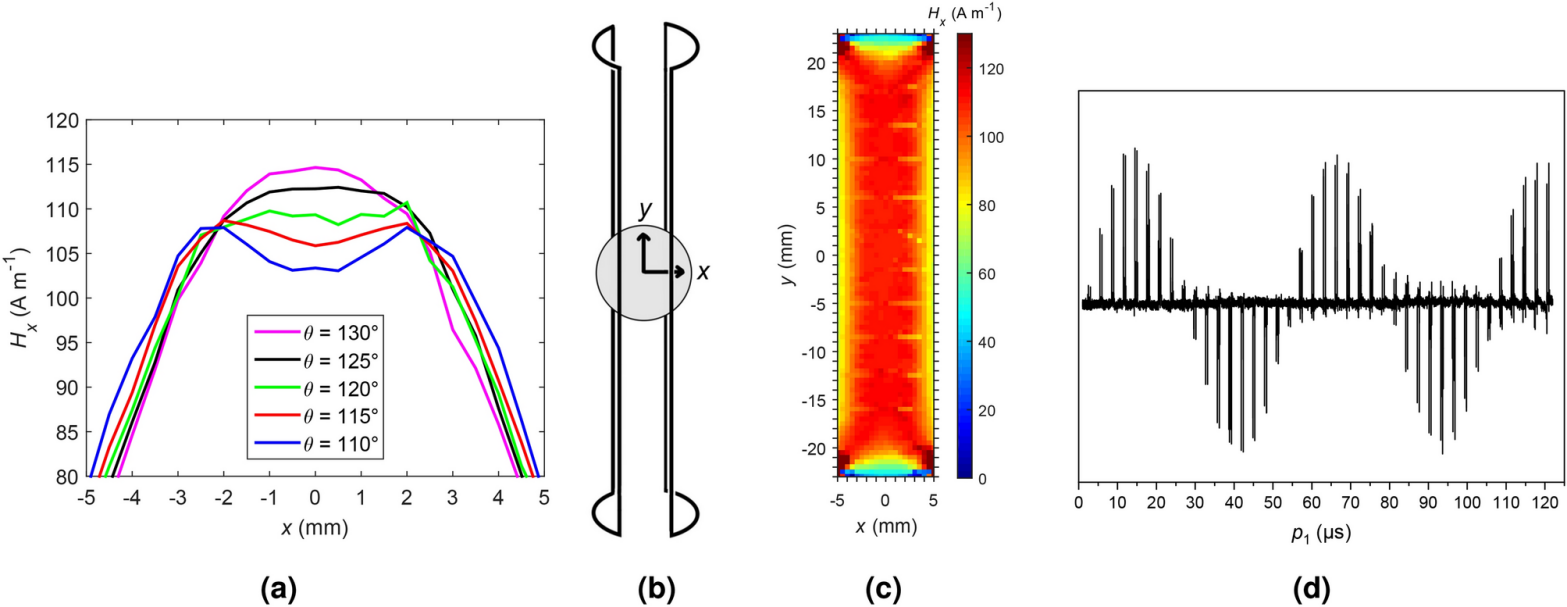
(**a**) Simulated magnetic flux densities $H_x$ for saddle coils with different opening angles *θ*, along the *x*-profile that corresponds to the direction of the generated magnetic RF field. (**b**) Schematic drawing of the saddle coil with an opening angle of 120$^\circ$ facing the *xy*-plane. The disk-shaped sample is schematically depicted as a grey circle. (**c**) Simulated $H_x$ for the central *xy*-plane of the saddle coil (*z *= 0 mm) with an opening angle of 120$^\circ$ . (**d**) $^{19}$F NMR nutation curve for different pulse lengths $p_1$ of LP30 (1.0 M LiPF$_6$ in ethylene carbonate (EC) and dimethyl carbonate (DMC) with EC/DMC=50/50 (v/v)) measured using the saddle coil with three windings per wing at 3960.4 G with an RF power of 30 W and $N_\textrm{scans}=16$ each.

In other works utilizing saddle coils for DNP often an opening angle of 90$^\circ$ was chosen^28,39–41^. However, according to other sources, the optimal geometry for a saddle coil to generate a uniform ***B***$_1$-field consists of a height with twice the value of the diameter and an opening angle of $120^\circ$^42,43^. To account for the specific coil geometry parameters, including the height/diameter ratio of 4.5, finite element method (FEM) simulations were performed. It was aimed for good field homogeneity both in *x*- and in *y*-direction, since the battery was aligned in this plane. While deviations were low along the *y*-direction due to symmetry reasons, the oscillating RF field amplitude $H_{x}$ varied in *x*-direction for different opening angles (Fig. 2a).

The best magnetic field homogeneity in the *x*-direction of the oscillating magnetic field was obtained for the coil with an opening angle of 120$^\circ$, with a flat plateau over an *x*-range of roughly ±2 mm. For visualization, the amplitude values $|H_x| = \sqrt{\textrm{Re}(H_x)^2 + \textrm{Im}(H_x)^2}$  were plotted for the *xy*-plane that intersects the central point of the coil (Fig. 2b, c). The simulations of this plane reveal that the magnetic flux density remains constant over a range of ±18 mm along *y*. Towards the edges, a decrease of the magnetic flux density is observable due to the wire aligned in the *xz*-plane.

As it provided the best field homogeneity, an opening angle of 120$^\circ$ was chosen for the practical implementation of the coil. The coil was built from insulated copper wire with a diameter of 0.22 mm and was held in position on a cylindrical glass tube using Scotch Magic Tape (3M), because it was found experimentally that this tape does not show an EPR signal. In contrast, it contributed to a $^1$H NMR background signal, which was subtracted in the respective spectra.

In addition to the opening angle of the saddle coil, also its number of windings was optimized for a better signal-to-noise ratio (SNR). It was assumed that more windings would not significantly influence the optimum opening angle with respect to field homogeneity. The best results were obtained with three windings per wing of the saddle coil. More windings did no longer allow us to set the desired resonance frequencies due to the increased coil inductivity.

To test the coil performance, a nutation curve was recorded (Fig. 2d)^44^. For this purpose, $^{19}$F NMR measurements were conducted on a LP30-sample (1.0 M LiPF$_6$ in ethylene carbonate (EC) and dimethyl carbonate (DMC) with EC/DMC=50/50 (v/v)), which provided narrow $^{19}$F NMR signals. The signal intensity of the second maximum at a flip angle of 450$^\circ$ corresponded to roughly 93 $\%$ of the signal of the first maximum at 90$^\circ$. This indicates a high coil homogeneity, which is consistent with the numerical simulations.

#### Microwave irradiation

**Fig. 3:**
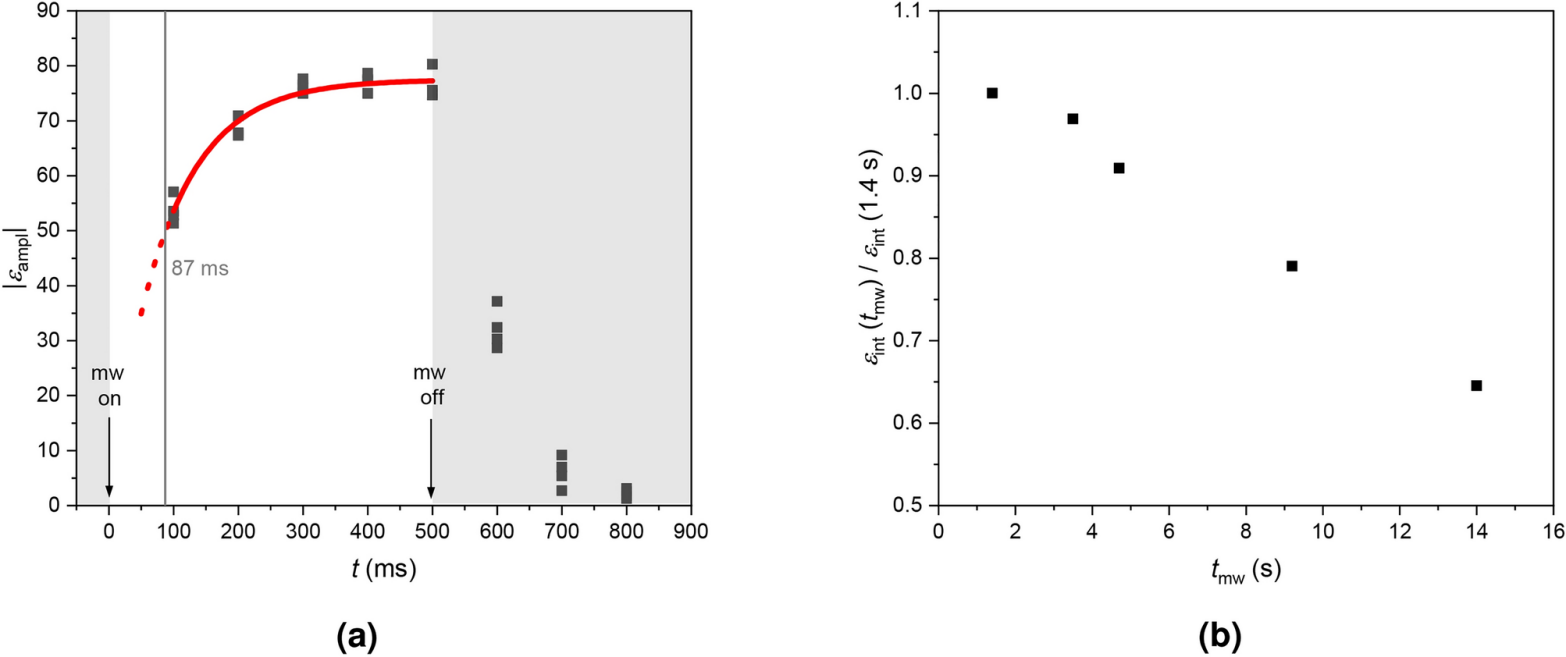
(**a**) Amplitude enhancement $|\epsilon _\textrm{ampl}|$ of the $^1$H NMR spectrum of 33 mM TEMPOL (aq) as a function of the time *t*. The sample was irradiated for 0.5 s. The amplitude enhancement was fitted (solid red line) and extrapolated to lower values (dotted red line). (**b**) Effect of mw irradiation times longer than 0.5 s on the integral enhancement as a function of the mw irradiation time $t_\textrm{mw}$. The integrals of the DNP-enhanced $^1$H NMR spectra were normalized by dividing them by the integral at $t_\textrm{mw}$=1.4 s. A mw power of 7.9 W was applied and $N_\textrm{scans}=1$ were acquired.

Since the maximum mw power of the EPR spectrometer was limited to 150 mW, an external mw source on an amplifier board providing a maximum output power of 15.8 W was installed to achieve a saturation of the conduction EPR transition that would be sufficient for DNP experiments (see Methods section for implementation details). First experiments were conducted with the well-studied^45^ 4-hydroxy-2,2,6,6-tetramethyl-piperidin-1-oxyl (TEMPOL) radical in aqueous solution. From the increasing amplitude enhancement $\epsilon _\textrm{ampl}$ with increasing mw duration (Fig. 3a) a DNP buildup time of 87 ms was estimated via an exponential fit. However, continuous sample irradiation for more than 0.5 s at such a high power level induced considerable sample heating. This was demonstrated in optimization experiments on TEMPOL (aq) that showed how the maximum enhancement was followed by a steady decrease as the mw irradiation time $t_\textrm{mw}$ prior to NMR acquisition was increased further (Fig. 3b).

After $t_\textrm{mw}=14$ s, 65 % of the initial enhancement remained. Due to the excitation of the rotational modes of water, sample heating is occurring upon longer mw irradiation times. The resulting decrease of the DNP enhancement may be caused by different factors. The efficiency of the ODNP process may be reduced due to the ZQ and DQ rates approaching each other, making the polarization transfer less efficient. Moreover, reduced relaxation times upon sample heating are expected to result in weaker saturation of the EPR resonance and a stronger leakage of nuclear polarization. While these measurements were conducted with aqueous samples, similar effects are expected for battery cells containing liquid electrolyte with a high dielectric constant. In addition, eddy currents from overlap of metallic battery components with the electric field part of the mw radiation cause further heating. To mitigate heating issues, a relay for pulsing the mw irradiation was incorporated into the setup, and mw irradiation was synchronized with the NMR acquisition. The mw irradiation was applied for a duration of 0.5 s and started 0.4 s prior to the NMR acquisition. To acquire several scans, a repetition interval of 10 s was chosen, resulting in a duty cycle of 5 %.

### DNP-enhanced $^{7}\textrm{Li}$ NMR signal from electrochemically deposited lithium

**Fig. 4:**
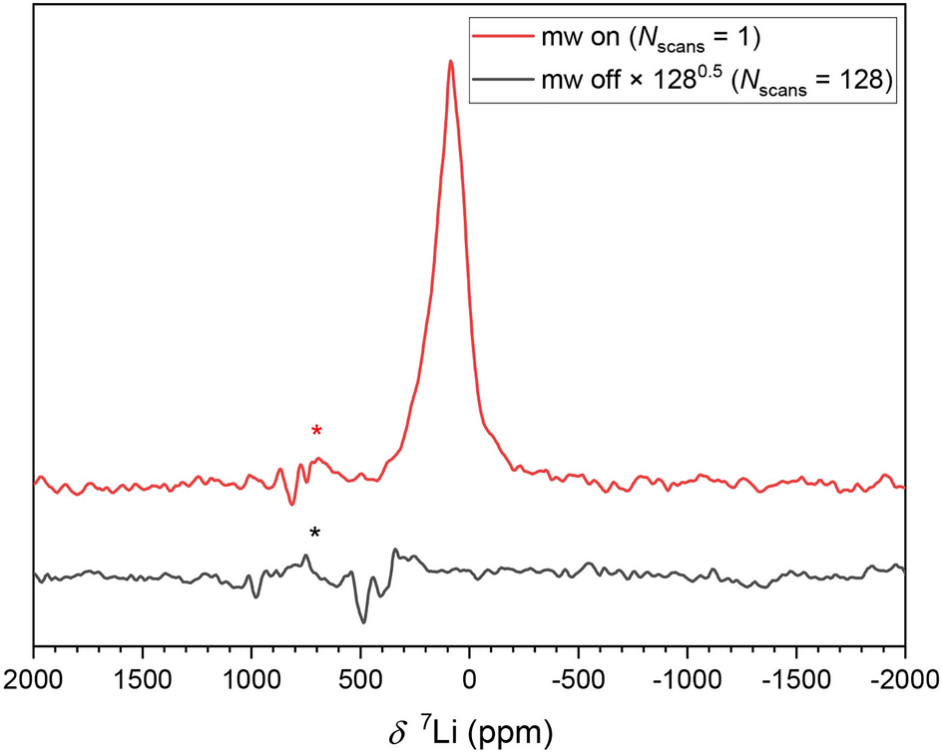
DNP-enhanced $^{7}\textrm{Li}$ NMR spectra of a Cu disk with electrochemically deposited Li without (black, $N_\textrm{scans}$=128) and with (red, $N_\textrm{scans}$=1) mw irradiation at 15.8 W and a mw frequency of 9.299 GHz. The spectra were recorded at 3317.9 G with an RF frequency of 5.49 MHz. Interference signals coupled in from outside are denoted by an asterisk.

A DNP-enhanced $^{7}\textrm{Li}$ signal of the Cu electrode and separator with deposited lithium was already detectable for the number of NMR scans $N_\textrm{scans}=1$ (Fig. 4). In contrast, also the spectrum recorded with $N_\textrm{scans}=128$ does not show a lithium signal with thermal spin polarization. From the SNR of 38.7 and $N_\textrm{scans}$ recorded without mw irradiation a minimum enhancement of $\epsilon \approx 400$ was estimated.

The chemical shift of lithium metal is normally approximately at 250 ppm, primarily caused by the FC interaction part of the Knight shift^23,37^. Here, the chemical shift is significantly smaller with a maximum at around 80–90 ppm. This could be traced back to the partial saturation of the electron resonances, which reduces the Pauli paramagnetic moment and, as a result, also reduces the Knight shift^23,38^. Additional line broadening is caused by inhomogeneities of the mw saturation, caused by shielding effects, different relaxation times or the skin effect, which occur to a different extent in different sample regions.

**Fig. 5:**
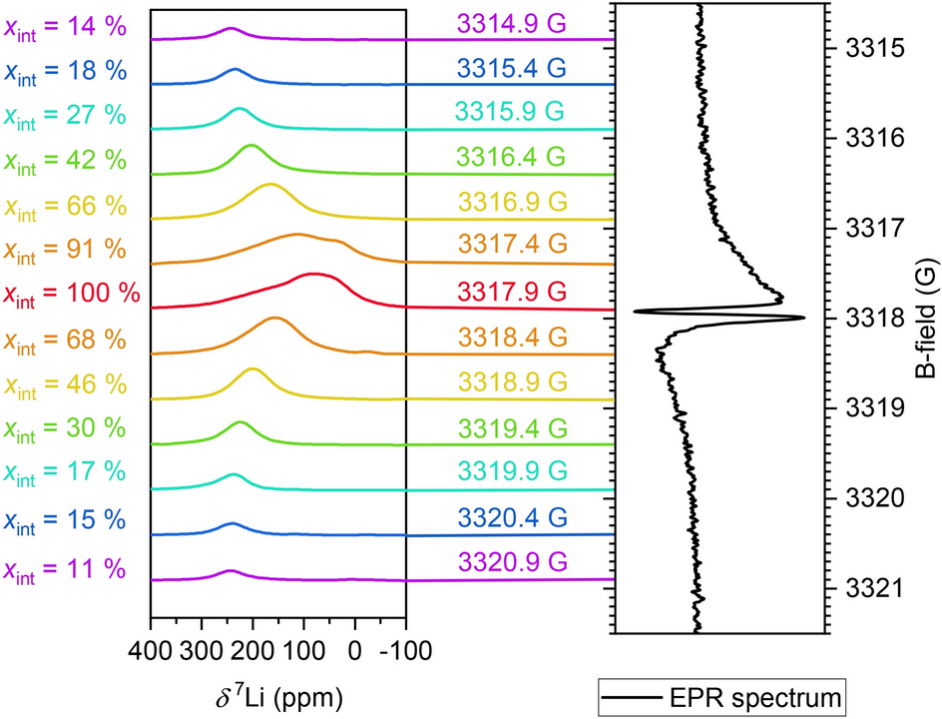
(Left) DNP-enhanced $^{7}\textrm{Li}$ NMR spectra of the Cu disk with electrochemically deposited Li at different magnetic fields with $f_\textrm{RF}$=5.49 MHz and $N_\textrm{scans}=16$. (Right) EPR spectrum of the same sample, recorded under non-saturating conditions. The DNP-enhanced NMR spectrum as well as the EPR spectrum acquisition were conducted with a mw frequency of 9.299 GHz and an EPR *Q* factor of 100.

To investigate the Knight shift hypothesis, the DNP enhancement was further investigated by varying the magnetic field. The acquisition and the processing parameters were chosen analogously as before, but with $N_\textrm{scans} = 16$. The $^{7}\textrm{Li}$ NMR spectra were measured at the same NMR resonance frequency ($f_\textrm{RF} = 5.49$ MHz) but with different magnetic fields, resulting in the spectra depicted in Fig. 5. The chemical shift of each spectrum was corrected for the respective magnetic field and each integral was determined as a percentage $x_\textrm{int}$ of the integrated spectrum obtained at 3417.9 G.

Over a range of 6 G, which is broader than all of the EPR linewidths observable in the EPR spectrum, a significantly enhanced signal can be observed in the $^{7}\textrm{Li}$ NMR spectrum. The $^{7}\textrm{Li}$ chemical shift of the signal increases from approximately 80 ppm (3417.9 G) to approximately 240 ppm (3414.9 G and 3320.9 G). This verifies the Knight shift hypothesis, since the smallest Knight shift is observed for exciting the EPR resonance centrally. Moreover, the calculated $x_\textrm{int}$ is largest for magnetic field strengths close to 3417.9 G.

The peak-to-peak EPR linewidths of two observable overlapping signals were 0.45 G and 0.07 G. These are on the same order of magnitude as reported for mossy and dendritic lithium, respectively^34^. While the narrower of the two signals is clearly distinguishable in the first derivative EPR spectrum, it is estimated to amount to less than 1 % of the double integrated signal amplitude. Therefore, corresponding $^{7}\textrm{Li}$ NMR signals are not expected to be observable. When assuming a homogeneous line for the broad signal, the corresponding transverse relaxation time can be estimated as $T_2 \approx 150$ ns. By further assuming $T_1 \approx T_2$, from the variation of the Knight shift and, independently, of $\epsilon$ as a function of the external field, the amplitude of the mw field used for DNP could be estimated to be approximately 1 G.

Due to saturation broadening at high mw power, DNP enhancement over a magnetic field range larger than the EPR linewidth was achieved. This could be further affected by the quality of contact between lithium metal and the copper electrode. According to Vigouroux *et al.*, lithium in very good contact with copper exhibits a broad EPR signal of roughly 5 G^46^. In the current work, such an EPR signal could be part of the baseline, but still affect the DNP enhancement, albeit the main DNP-enhanced signal was consistent with the 0.45 G wide EPR line. However, there is an indication of a second Knight-shifted species that is observable at about 3317.4 G. It is only discernible from the main DNP-enhanced $^{7}\textrm{Li}$ resonance over a narrow field range, which implies a lower local mw amplitude, yet somewhat longer relaxation time constants as the Knight shift is not reduced. The same effect could also explain the very minor downfield shoulder of the EPR spectrum measured at 3317.4 G. These observations could be linked with the hypothesis in our previous work that suggested differences in Li layers above a Cu foil^36^. As a possible explanation the different degree of contact between electrochemically deposited lithium and copper could be identified. A better contact between Li and Cu may be linked with an increased mw field^47^, yet reduced contact may favor longer relaxation time constants^46^. Therefore, the correlation experiment between the external magnetic field and the Knight shift would provide a method to distinguish between loosely and well contacted lithium. The distinction of two different lines in the DNP-enhanced $^{7}\textrm{Li}$ NMR spectrum indicates that this contact is not varying in a continuous manner from poor to good, but that discrete states may be assumed.

**Fig. 6:**
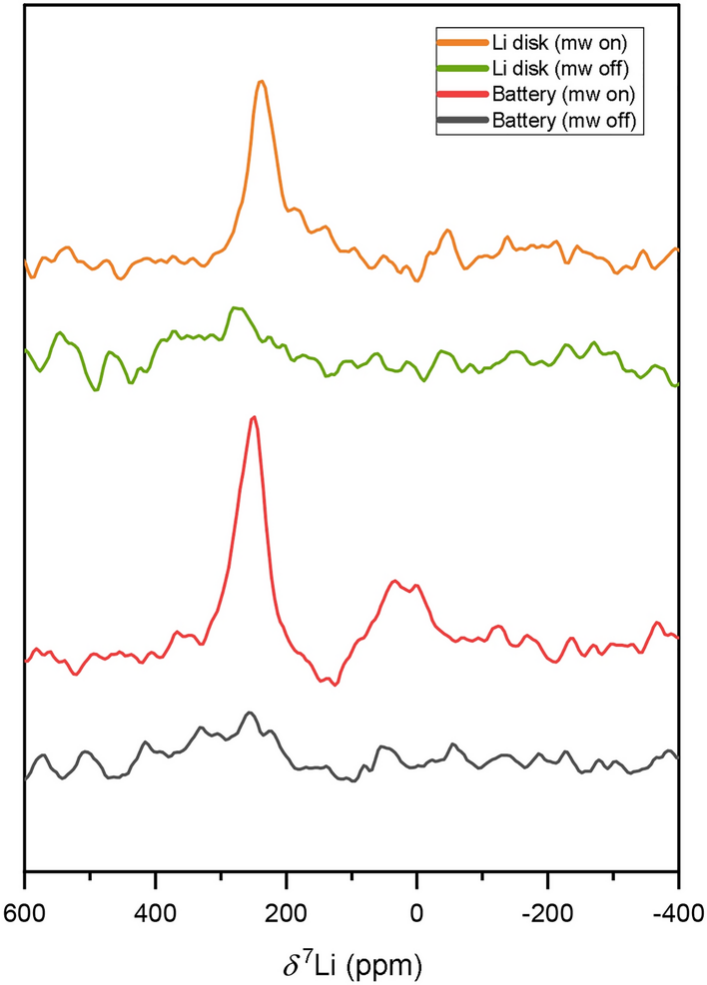
$^{7}\textrm{Li}$ NMR spectra of an assembled Li vs. Cu half cell without (black) and with 15.8 W mw irradiation (red). The spectra were measured at 3361.9 G with $f_\textrm{RF}=$5.563 MHz, $f_\textrm{mw}=$9.413 GHz, and an EPR *Q* factor of 1300. Also a pure lithium disk was measured without (green) and with (orange) 15.8 W mw irradiation. The pure lithium sample was measured at 3342.5 G with $f_\textrm{RF}=$5.531 MHz, $f_\textrm{mw}=$9.359 GHz, and an EPR *Q* factor of 1000. All spectra were recorded with $N_\textrm{scans}=128$.

Further investigations were conducted with a complete Li vs. Cu half cell that was not disassembled after Li was deposited on Cu. $^{7}\textrm{Li}$ enhanced NMR spectra were obtained showing a broad signal centered at around 250 ppm and a second signal with a lower intensity centered around 0 ppm (Fig. 6).

When the mw irradiation was switched on, an amplitude enhancement of roughly $\epsilon _\textrm{ampl} \approx 6.8$ was observed for the signal at approximately 249 ppm. The integral enhancement was not calculated due to the difficulties of distinguishing between signal and noise. To investigate whether the lithium electrode instead of deposited lithium was causing the signal, the measurement was repeated with an equally sized piece of lithium. It also exhibited a signal in the DNP-enhanced $^{7}\textrm{Li}$ NMR spectrum with a maximum intensity at 237 ppm. Its amplitude enhancement amounted to $\epsilon _\textrm{ampl} \approx 3.1$, which is smaller than the amplitude in the $^{7}\textrm{Li}$ NMR spectra of the battery. This could be a result of signal broadening, which is consistent with better saturation of the electron resonances in the lithium disk, as the maximum intensity is shifted to smaller chemical shift values compared to the battery. In addition, there could also be a contribution of lithium deposited on Cu to the signal around 249 ppm, yet an unambiguous distinction of the origin of the signal could not be made. Experiments using other anode materials to identify the source of the observed signal are ongoing. Nevertheless, the DNP enhancement observed in the spectra depicted in Fig. 6 was relatively low compared to the electrochemically deposited lithium measured only on copper (Fig. 5). The absence of a significant Knight shift reduction for the $^{7}\textrm{Li}$ resonance of metallic Li indicates a reduced mw field amplitude as the main reason for the reduced enhancement. This may be caused by the larger sample thickness compared with the disassembled Cu plate. Moreover, coupling of the mw field into the gap between the Cu and the Li electrode may be inefficient with the chosen resonator. Finally, it may be possible that a mode conversion occurred in the resonator by the presence of the Cu plate, which would be most efficient with a highly conducting metal plate.

The signal around 0 ppm was only observed for the battery, but not for the lithium plate. This indicates that there was a polarization of $^{7}\textrm{Li}$-nuclei in LiPF$_6$ or the SEI in close proximity to metallic lithium.

### DNP-enhanced $^1$H signal

**Fig. 7:**
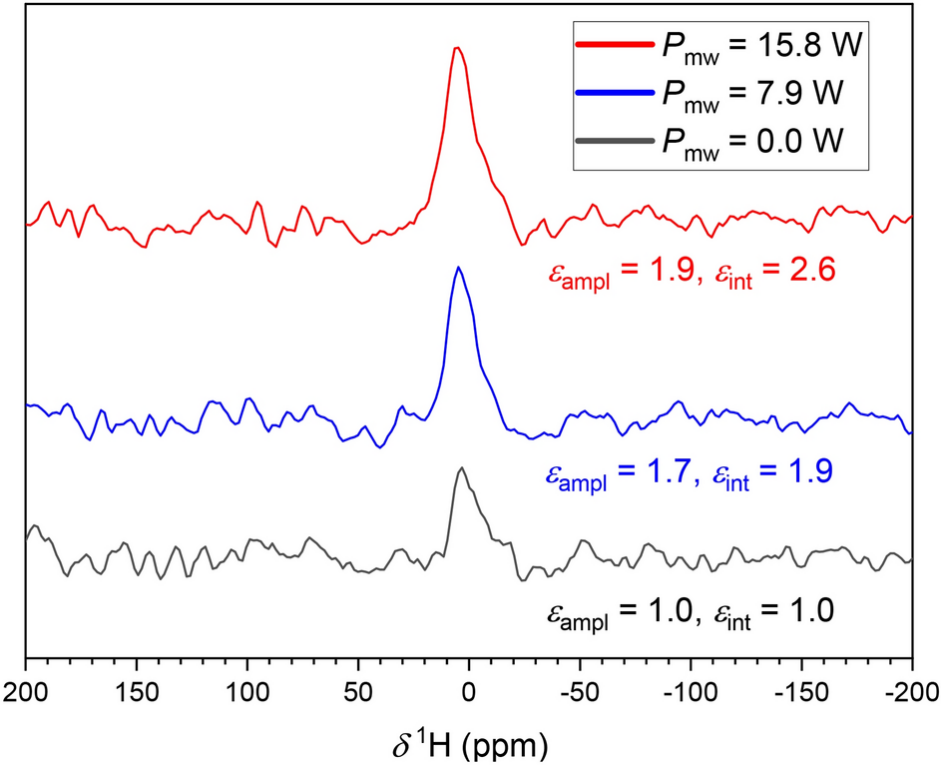
$^1$H NMR spectra of electrochemically deposited lithium wetted with electrolyte measured at 3415.1 G, an RF frequency of 14.54 MHz, a mw frequency of 9.564 GHz, $N_\textrm{scans}=32$, and an EPR *Q* factor of 600. The spectra were recorded without mw irradiation (black), with a mw power of 7.9 W (blue), and with a mw power of 15.8 W (red).

$^1$H NMR measurements of the disassembled sample containing electrochemically deposited lithium using LiPF$_6$ in propylene carbonate (PC) were conducted with and without mw irradiation with $N_\textrm{scans}=32$ each. To compare the observed signals, the corresponding background spectra were subtracted (Fig. 7, see Supplementary Material (S1) for spectra before background subtraction).

Upon turning on the mw irradiation with a power level $P_\textrm{mw}$ = 7.9 W, a positive signal enhancement was observed, which intensified with $P_\textrm{mw}$ = 15.8 W. The positive enhancement is in agreement with the findings of Hope *et al.*^23^ They observed DNP enhancement of $^1$H NMR spectra upon mw irradiation of conduction EPR resonances from lithium microstructures formed during cycling, which they traced back to solvent molecules trapped within pores of the SEI and/or small organic lithium carbonates^48^. Heat-induced degradation of the sample was excluded as a cause of the increasing signal by a subsequent control experiment (see also S1). 

Due to the low magnetic field strength and the lack of magic angle spinning (MAS), no detailed distinction of the environment of the measured protons was possible with our setup. Despite this, the $^{1}\textrm{H}$ NMR line width indicates that the signal was caused by a mobile proton-containing species, such as trapped liquid electrolyte or a soft SEI contribution. Since enhancement is mediated by conduction electron spin from Li metal, the local signal enhancement is likely to exceed the observed overall enhancement. The mechanism for the polarization enhancement of $^1$H is not yet fully understood, but two possible origins have been reported^23^. The $^1$H nuclei could be either directly polarized due to their immediate proximity to the lithium surface, followed by spin diffusion. Alternatively, there could be cross-relaxation between $^1$H-nuclei and already polarized $^{7}\textrm{Li}$-nuclei via a nuclear Overhauser effect.

## Conclusion

A setup for DNP-enhanced NMR measurements was developed based on an X-band EPR spectrometer and a low-field NMR console. A saddle coil geometry was optimized using FEM simulations, and a coil opening angle of 120$^\circ$ gave a very homogeneous RF field while showing good compatibility with the mode of the mw resonator. The signal-to-noise ratio was maximized with three windings per wing. An external mw source in combination with a 15.8 W amplifier was employed for DNP operation, while EPR spectra of the same sample were acquired with the bridge of the EPR spectrometer. To minimize sample heating, a relay was incorporated to allow pulsed DNP. 

$^{7}\textrm{Li}$ NMR of electrochemically deposited lithium on a copper disk was only detectable using DNP, with an estimated minimum enhancement of $\epsilon \approx 400$. The signal of metallic Li exhibited a maximum at around 80 ppm, which is lower than otherwise common due to the reduced Knight shift, resulting from the partial saturation of the Pauli conduction electron paramagnetic resonances. This was verified by varying the static magnetic field. Using an estimate of the EPR relaxation time constants based on the conduction EPR line width, the mw field amplitude was determined.

Variations of the Knight shift were consistent with a dominant metallic Li species that showed a homogeneous conduction EPR spectrum, yet with a substantial secondary component, indicating the presence of two lithium species with different quality of contact with the copper surface.This previously unattainable insight highlights the potential of combining EPR with DNP-enhanced NMR for distinguishing lithium species at the copper interface.

DNP-enhanced $^1$H NMR measurements of samples that were prepared by electrochemical Li deposition in a PC based electrolyte revealed a proton signal enhancement of $\epsilon _\textrm{int}=2.6$ and $\epsilon _\textrm{ampl}=1.9$, which was traced back to polarization transfer from metallic lithium to $^1$H-nuclei of neighboring solvent molecules at the interface.

Compared to high-field MAS DNP experiments, higher DNP enhancement may be achieved. On the other hand, the presented low-field DNP setup lacks the resolution to distinguish different species with similar chemical shifts, such as lithium moieties in the SEI or in the electrolyte. Moreover, the presented setup is currently limited to single-resonance experiments. Further hardware development would be required to establish the prerequisites for double-resonance experiments, such as cross polarization. However, this method lays the foundation for further experiments that correlate DNP-enhanced NMR measurements at different magnetic field strengths with EPR experiments. By improving the mw switching hardware as well as the synchronization between mw irradiation and the acquisition of NMR spectra, DNP-enhanced $^{7}\textrm{Li}$ NMR spectra could be recorded that acquire NMR spectra shortly after the mw irradiation, enabling a correlation not only with DNP buildup times, but also with relaxation times after turning off the mw field as an additional dimension. This could help distinguishing between polarization enhancement via a direct nuclear Overhauser mechanism or via spin diffusion, which occur on different time scales, to shed further light on environments and distances or depth profiles of DNP-enhanced species. The DNP-enhanced $^1$H NMR spectra could potentially be used to determine the thickness of the inorganic inner SEI layer, since the thinner the SEI, the larger the $^1$H enhancement resulting from organic compounds in the outer SEI layer. To approach this goal, the synthesis of reference samples with well characterized SEI thickness and composition would be required.

Eventually, it was demonstrated that the presented setup is suitable for future investigations of working batteries using DNP-enhanced NMR, including *in operando* experiments. With the presented setup, this was limited by the loss of the EPR resonator mode caused by damping effects from polar electrolyte solvents.

## Methods

### DNP setup

A Kea$^2$ NMR spectrometer (5–50 MHz, Magritek GmbH) was used to provide the RF pulses and to detect the NMR signal. It was powered by an AC to DC converter (EPA150-24-15, Powersolve Electronics Ltd, 24 VDC and 6.25 A) and controlled by the software Prospa (V3.39), which ran on a connected laptop. Its RF output was connected to the probe via a coaxial RF cable (length: 1.5 m, 50 $\Omega$ impedance), featuring an electrically conductive cable sheath that was used for grounding. All NMR measurements were conducted using the magnet of a Bruker ElexSys E540 X-band spectrometer. To adjust the resonance frequency, the NMR coil was integrated into a resonant circuit that was based on a capacitive current divider configuration (see the circuit diagram on the right in Fig. 1). Except for the coil, the resonant circuit was placed in an aluminum box (Camdenboss 5000, dimensions 120 $\times$ 66 $\times$ 40 mm IP54). On one side of the aluminum box, an SMB bulkhead (inner pin male) was placed to connect the external RF source to the resonant circuit. From the inside of the aluminum box, insulated copper wire (CUL 100/0,35, Block) was soldered to the SMB bulkhead, while another piece of copper wire was attached to its casing for grounding. Both copper wires were attached via a connector (Jacks, 1.0 mm, type MC LS1-B) to a pin (Plug pins, 1.0 mm, for PCBs, type MC S1-F) on a copper circuit board (RE520 from Roth Elektronik). The circuit board was an exchangeable module to accommodate different capacitors for varying the resonance frequency. A combination of ceramic capacitors (Velleman group K/CAP1, 10 pF to 220 nF) and trimmer capacitors (e.g. Trimmer 31659 from Reichelt) were soldered (STA 611 TC 1,0 from Stannol) on the circuit board. The capacitor combinations relevant for this work are listed in Table 1 with the corresponding resonance frequencies.

**Table 1:** Variable and fixed capacitors used for the NMR resonant circuits, along with the achieved NMR *Q* factor. The nuclei of interest are listed with their RF frequencies for the discussed experiments.

Nucleus	RF frequency (MHz)	NMR *Q* factor (inside resonator)	(Variable capacitor) + fixed capacitor
$^1$H	14.540	130	Match: (4.5 – 70 pF)
			Tune: (4.5 – 70 pF) + (1.5 – 5 pF)
$^{7}\textrm{Li}$	5.490, 5.531	70	Match: (4.5 – 70 pF) + 100 pF
			Tune: 2×(4.5 – 70 pF) + 367 pF

The trimmer capacitors could be adjusted for matching and tuning through holes drilled in the aluminum box. Two low noise cables (LN5002, length: 10 cm) were used to connect the aluminum box to the saddle coil. The outer shielding mesh of the cables was connected to common ground. To avoid wobbling and hence instabilities of the resonant circuit, the cables were stabilized with cable grommets (PFA Swagelok Tube Fitting PFA-220-6). To minimize interfering RF signals, aluminum foil was wrapped around the probe and was connected to the electrically conductive RF cable sheath.

### Finite element method simulations

The program EMPro 2020 was used to perform FEM simulations. According to the experimental conditions, the simulated coil diameter was set to 1 cm and the coil height to 4.5 cm. The simulated rectangular copper wire cross section was 0.1 mm $\times$ 0.1 mm and a current of 1 A was simulated with a frequency of 10 MHz. The magnetic flux density was simulated for an opening angle of $\theta$ = 120$^\circ$. An imaginary and a real component of $H_x$ were obtained representing the phase shift of the RF signal. Here we are only interested in the amplitude $|H_x| = \sqrt{\textrm{Re}(H_x)^2 + \textrm{Im}(H_x)^2}$, which was calculated and visualized as a heat map using Matlab 2015a.

### Microwave source

The mw setup, including mwgenerator and amplifier board, was fully described in a previous publication^39^. In this work, instead of using the low-power mode of the amplifier board for tuning, the EPR spectrometer with the Xepr software was used for tuning the resonator. Moreover, the resonator utilized by Überrück *et al.* was replaced by a Bruker ER 4103TM resonator. After tuning, the high-power mode of the amplifier board was used for the DNP experiments. The mw frequency and amplitude could be set on the mw generator (N5173B, Keysight Technologies, maximum power of 80 mW). The mw was amplified by 42 dB with a maximum output power of 15.8 W in a range of 9.3 to 9.8 GHz facilitating the equipment described by Überrück et al.^39^ Using a coaxial switch (Teledyne Failsafe SPDT Coaxial Switch CCR-33S30-T), the mw path could be toggled after the mw generator either into the amplifier board or into a 50 $\Omega$ load for providing mw pulses instead of continuous irradiation (Fig. 1). Without an external control voltage at the logic input 1, the mws were directed to the output channel that was terminated with a mw blocker exhibiting a resistance of 50 $\Omega$. Upon application of an external pulse of 5 V at the logic input 1, the relay was switched to the channel connected to the amplifier board via a coaxial cable (SMA plug to SMA plug, 50 $\Omega$, $\sim$ 18 GHz, conformable). The actuation voltage required for switching the relay was provided by an additional power supply (Voltcraft PPS-16005, 12 V, 0.1 A). For controlling the relay, a pin of an Elegoo Uno R3 Controller Board was connected to the logic input of the relay. Pulsing of the mws was initialized by pressing an activation button on the breadboard. The Uno R3 was programmed using Arduino IDE 2.2.1 to provide pulses lasting for 0.5 s, which were repeated every 10 s.

### DNP workflow

First, the sample was centered vertically in the glass tube. Then the saddle coil was positioned around it and it was inserted into the Bruker ER 4103TM resonator. By stabilizing the waveguide of the EPR resonator with two large plastic screws, it was held in position. The correct orientation of the saddle coil was ensured visually, while the insertion depth into the resonator was controlled using the tuning mode of the EPR spectrometer to obtain a dip with maximum *Q* factor. The EPR *Q* factors provided in this work were read out via the Xepr software at 33 dB attenuation. To prevent drifts during the experiment, the EPR magnet was turned on at least 1 h in advance. Then an EPR spectrum was recorded by utilizing the X-band mw bridge to identify the conditions for partially saturating the electron resonance. Afterwards the modulation cable and the mw bridge were disconnected and the amplifier board connected to the resonator. The RF circuit was tuned and matched using an external spectrum analyzer (Rigol DSA815 Spectrum Analyzer LXI 9 kHz–1.5 GHz). Depending on the resonance frequency of the mw resonator, the corresponding static magnetic field was set using the Xepr software (2.6b.167 (2017-Jul-17), Bruker). The frequency of the mw generator was set according to the frequency the resonator was tuned to.

### EPR measurement

The EPR spectrum was recorded using the X-band mw bridge with a mw frequency of 9.299 GHz and a mw power of $${75.18}{\ \upmu \hbox {W}}\:($$power attenuation of 33 dB$$)$$. 8 scans were recorded with an EPR *Q* factor of 100 and a conversion time of 60 ms. The modulation was performed with a frequency of 100 kHz and a modulation amplitude of 0.02 G.

### NMR measurements

The NMR measurements, except for the nutation curve, were conducted by utilizing a Kea$^2$ console (Magritek), which was controlled by a laptop with the software Prospa (V3-39). Data processing, including SNR determination, was done using MestReNova (version 14.2.3-29241).

Single-pulse experiments were conducted for all shown $$^1$$H NMR spectra with an RF power of 22 W and a pulse length of 14 $${\upmu \hbox {s}}$$. For single-pulse $$^{7}\textrm{Li}$$ NMR experiments an RF power of 44 W and pulse lengths of 14 $$\:{\upmu \hbox {s}}$$ $$($$Fig. 4 and 5$$)$$ and 16 $${\upmu \hbox {s}}\:($$Fig. 6$$)$$ were used. For $$^1$$H NMR spectra, a background spectrum was recorded and subtracted from the respective measurement. $$^{7}\textrm{Li}$$ NMR spectra were referenced to $${\textrm{Li}_2\textrm{SO}_4}\:($$aq$$)$$ at 0 ppm and $$^{1}$$H NMR spectra to $$\hbox {H}_{2}\hbox {O}$$ at 4.8 ppm. The $$^1$$H NMR spectra were apodized exponentially with a time constant corresponding to 50 Hz line broadening (Fig. 7), and the $$^{7}\textrm{Li}$$ spectra were apodized exponentially with a time constant corresponding to 200 Hz (Fig. 4 and 5) and 100 Hz (Fig. 6). For plotting, the spectra were exported to OriginPro 2021b (OriginLab Corporation, Northampton, MA, USA). In this work, the signal enhancement is provided as the amplitude ratio $$\epsilon _{\textrm{ampl}}$$ or the integral ratio $$\epsilon _{\textrm{int}}$$ of the NMR signal.

The $^{19}$F NMR nutation curve of LP30 was measured with the console of a Bruker AvanceIII-HD spectrometer, using TopSpin 3.6.1, at the magnet from the EPR spectrometer. 16 scans were acquired per point with an RF power of 30 W.

### Sample preparation

#### TEMPOL (aq)

TEMPOL (4-hydroxy-2,2,6,6-tetramethyl-piperidin-1-oxyl (aq), 33 mM, 95 $\%$ purity, Sigma-Aldrich) was prepared under ambient atmosphere. It was filled into an EPR tube (Wilmad quartz (CFQ) EPR tubes, O.D. 2 mm, I.D. 1 mm, L 100 mm, fill height: 8 mm), which was positioned centrally in a glass tube with a diameter of 1 cm.

#### Battery preparation (Fig. 4 and 5)

Lithium was plated on copper in a Swagelok cell. The cell was assembled under argon atmosphere using a 7 mm disk of Li foil (0.1 mm thickness, 99.9 $\%$ purity, Onyxmet), a 7 mm disk of Cu foil (${10}{\ \upmu \hbox {m}}$ thickness, Evonik), and a glass microfiber separator (Whatman GF/C) of 12 mm diameter and 0.26 mm thickness, soaked with 200 ${\upmu \hbox {L}}$ of LP30 electrolyte (1.0 M LiPF$_6$ in EC/DMC=50/50 (v/v), battery grade, Sigma-Aldrich). Lithium was plated onto copper at a constant current density of ${2}{\ \hbox {mA}\, \hbox {cm}^{-2}}$ for 1 h. Afterwards, the Swagelok cell was disassembled under argon atmosphere. The Li foil as well as several layers of the separator were stripped off. Theremaining separator was cut to a diameter of 8 mm. The resulting sample, consisting of the Cu disk with plated Li and the attached separator, was used for DNP experiments. Prior to conducting the DNP-enhanced NMR measurements, the sample was stored under argon atmosphere in a glovebox for three days. For the DNP measurements, the sample was transferred to a quartz glass cell described by Niemöller *et al.*^11^ This cell was put into the glass tube, which exhibits a cylindrical geometry (diameter: 1 cm, height: 20.5 cm). It was sealed air-tightly using joint grease.

#### Battery preparation (Fig. 6)

The battery half-cell was prepared under argon atmosphere in a glovebox. It was built using a lithium (0.1 mm thickness, purity of 99.9 %, Onyxmet) and a copper electrode, which were punched out from the corresponding plates with a diameter of 7 mm. A separator (Whatman GF/C) with a diameter of 8 mm and 0.26 mm thickness was placed between the electrodes and 10 ${\upmu \hbox {l}}$ of LP30 (1.0 M LiPF$_6$ in EC/DMC=50/50 (v/v), battery grade, Sigma-Aldrich) was added onto the separator. The battery was placed in an *in operando* cell described by Niemöller *et al.*^11^ This cell was placed inside a battery holder as described in the previous paragraph and each electrode was connected to a platinum wire. At first, Li was plated and stripped at a current density of ${4}{\ \hbox {mA}\, \hbox {cm}^{-2}}$ for 50 min each. Afterwards, Li was plated at a current density of ${1.3}{\ \hbox {mA}\, \hbox {cm}^{-2}}$ for 3 h. Prior to conducting the DNP-enhanced NMR measurements, the battery was stored under argon atmosphere in a glovebox for nine days, which resulted in the battery cell drying out.

#### Electrochemical lithium deposition

For electrochemical lithium deposition, a copper and a lithium electrode were chosen. The copper electrode was cut from copper plate (1 cm $\times$ 3 cm) and the lithium electrode was prepared by wrapping lithium (0.1 mm thickness, 99.9 % purity, Onyxmet) around a copper wire. They were placed with a distance of roughly 2 cm in a beaker under argon atmosphere in a glovebox. The electrolyte LiPF$_6$ in PC (1.0 M, battery grade, Sigma-Aldrich) was added into the beaker with a fill height of roughly 2 cm. The electrodes were connected to a 3.6 V battery using copper wires, initiating the deposition of lithium onto the copper electrode. This process continued for approximately 2 h. A mixture of dead and deposited lithium, which was scraped from the copper electrode, was filled into an EPR tube with a diameter of 4 mm (Wilmad quartz (CFQ) EPR tubes) under argon atmosphere in a glovebox. The lithium stuck to the glass wall, and the remaining electrolyte settled at the bottom of the tube. The latter was removed using a needle and a syringe. After closing the tube with a cap, additional parafilm was wrapped around it. The tube was then aligned vertically within the glass tube, which was sealed using joint grease and parafilm.

## Electronic supplementary material

Below is the link to the electronic supplementary material.

Supplementary Material

## Data Availability

Data for this article, including the DNP-enhanced NMR measurements, the EPR spectrum, and the simulation results are available on Jülich DATA at 10.26165/JUELICH-DATA/FEI8NB.
