# Supplementary material for: Combined dynamic nuclear polarization and electron paramagnetic resonance at 0.34 T to investigate electrochemical lithium deposition on copper
Source: Sci Rep. 2025 May 26;15:18436. doi: 10.1038/s41598-025-01107-x (PMC12106791; doi:10.1038/s41598-025-01107-x)
Supplement: Supplementary file 1 — Supplementary Material [file 41598_2025_1107_MOESM1_ESM.pdf]

Supporting information for: Combined dynamic nuclear polarization and electron paramagnetic resonance at 0.34 Tesla to investigate electrochemical lithium deposition on copper

Vera Michaela Barysch[1,2,*], Beatrice Wolff[1], Matthias Streun[3], Peter Jakes[1], Peter Philipp Maria Schleker[1], and Josef Granwehr[1,2]

[1]Forschungszentrum Jülich GmbH, Institute of Energy Technologies (IET-1), Jülich, 52425, Germany
[2]RWTH Aachen University, Institute of Technical and Macromolecular Chemistry, Aachen, 52056, Germany
[3]Forschungszentrum Jülich GmbH, Institute of Technology and Engineering (ITE), Jülich, 52425, Germany
[*]v.barysch@fz-juelich.de

Pulsing and triggering of the mw irradiation

For the DNP experiments, the mw irradiation was pulsed using a relay that could switch between a channel connected to the sample and a channel connected to a 50 Ω load (see also Fig. 1). To control the relay, pin ~6 of an Elegoo Uno R3 controller board was connected to the logic input of the relay by extending the breadboard jumper wire with an additional wire (wire LIY 0.5 mm^2). Its end was soldered to the logic input 1 of the relay. The pulsing of the mws was initialized by pressing an activation button on the breadboard. To monitor the switching process, a red control LED was added to indicate when the relay passed the mw radiation to the sample. A photo of the Uno R3 with the breadboard connected is shown in Figure S1.

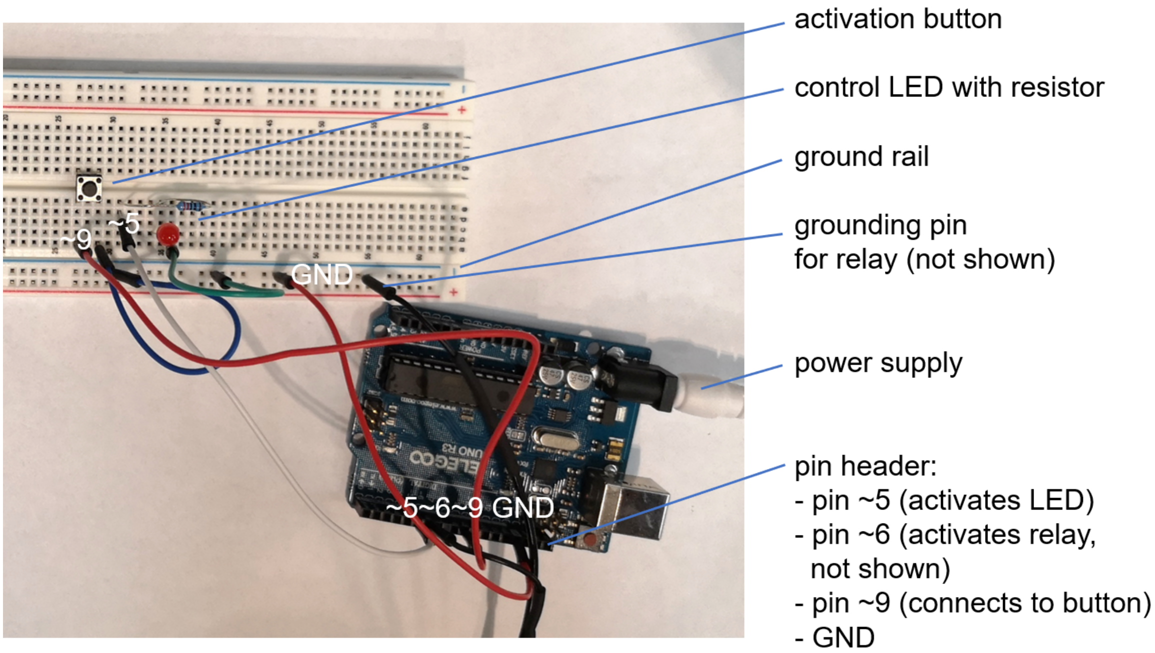

Fig. S1: Labelled photo of the Uno R3 with the corresponding breadboard used to switch the relay.

Pulsing was initialized by pressing the activation button. During pulsing, the relay and the

control LED were activated simultaneously via pin ~5 and pin ~6, respectively. The common terminal of the relay COM was connected to the ground rail as shown in figure S1. The Uno R3 was programmed using Arduino IDE 2.2.1 to deliver pulses of 0.5 s duration, repeated every 10 s.

To initialize the mw irradiation and the NMR acquisition, the activation button of the mw was pressed at the same time as the NMR acquisition was started by activating the button in the Prospa software. However, the NMR acquisition started with a delay. To account for this, the timing was systematically varied by setting an additional delay in the Arduino code. To identify the best delay, the TEMPOL (aq) enhancement was measured for different delays between activation of the mws and NMR acquisition, as shown in Fig. 3a). Assuming a human error of up to 0.1 s due to manual activation of the buttons, the NMR acquisition was set to 0.4 s after the start of the mw irradiation (see also Fig.3a)). Future improvements should include automatic triggering of the NMR acquisition, especially for time-dependent studies.

DNP-enhanced ^{1}H NMR spectra

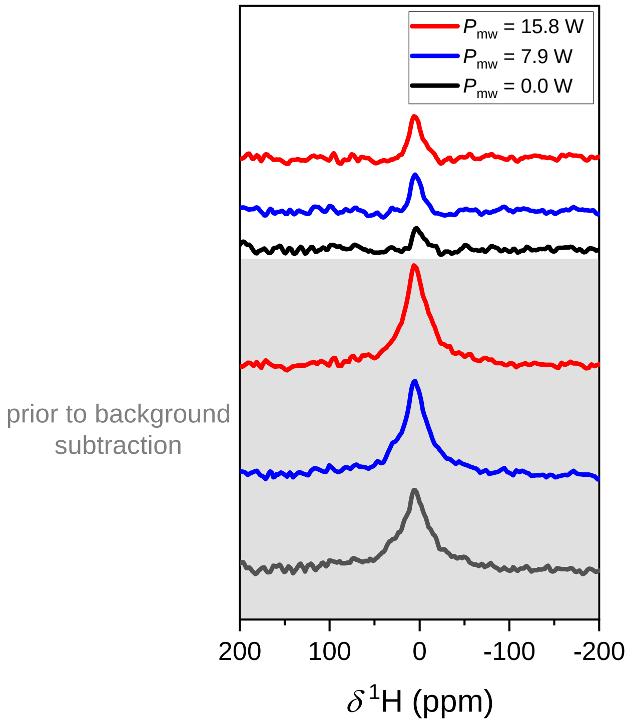

Fig. S2: ^{1}H NMR spectra of electrochemically deposited lithium measured at 3415.1 G, an RF frequency of 14.54 MHz, a mw frequency of 9.564 GHz, $N_{\text{scans}} = 32$, and an EPR Q factor of 600. The spectra were recorded without mw irradiation (black), with a mw power of 7.9 W (blue), and with a mw power of 15.8 W (red). The spectra are also depicted prior to background subtraction.

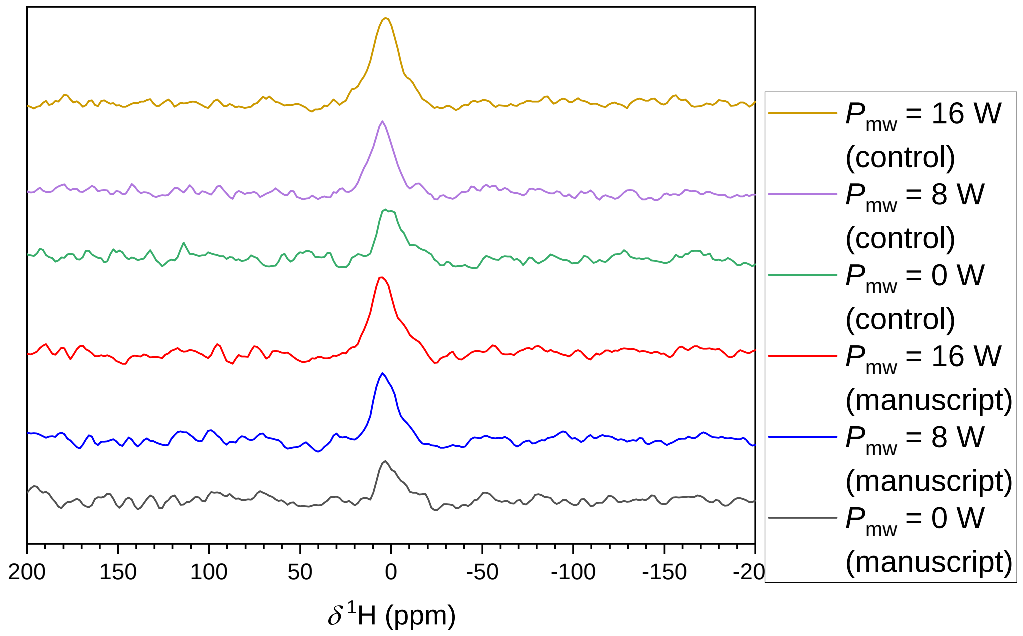

Fig. S3: Background-subtracted ^{1}H NMR spectra of electrochemically deposited lithium measured at 3415.1 G, an RF frequency of 14.54 MHz, a mw frequency of 9.564 GHz, $N_{\text{scans}} = 32$, and an EPR Q factor of 600. The spectra were recorded without mw irradiation (black), with a mw power of 7.9 W (blue), and with a mw power of 15.8 W (red). The experiment was repeated to check the reproducibility.
